# CCI-779 (Temsirolimus) exhibits increased anti-tumor activity in low EGFR expressing HNSCC cell lines and is effective in cells with acquired resistance to cisplatin or cetuximab

**DOI:** 10.1186/s12967-015-0456-6

**Published:** 2015-04-01

**Authors:** Franziska Niehr, Wilko Weichert, Albrecht Stenzinger, Volker Budach, Ingeborg Tinhofer

**Affiliations:** Translational Radiooncology Laboratory, Department of Radiooncology and Radiotherapy, Charité University Hospital, Berlin, Germany; German Cancer Consortium (DKTK), German Cancer Research Center (DKFZ) Heidelberg, Partner Site Berlin, Berlin, Germany; Institute of Pathology, University Hospital Heidelberg, Heidelberg, Germany; German Cancer Consortium (DKTK), German Cancer Research Center (DKFZ) Heidelberg, Partner Site Heidelberg, Heidelberg, Germany; Department of Radiooncology and Radiotherapy, Translational Radiooncology Laboratory, Charité-Universitätsmedizin Berlin, Charitéplatz 1, 10117 Berlin, Germany

**Keywords:** HNSCC, mTOR, EGFR, Temsirolimus, CCI-779, Cetuximab, Cisplatin, Resistance

## Abstract

**Background:**

The mammalian target of rapamycin (mTOR) signaling pathway plays a pivotal role in numerous cellular processes involving growth, proliferation and survival. The purpose of this study was to investigate the anti-tumoral effect of the mTOR inhibitor (mTORi) CCI-779 in HNSCC cell lines and its potency in cisplatin- and cetuximab-resistant cells.

**Methods:**

A panel of 10 HNSCC cell lines with differences in *TP53* mutational status and basal cisplatin sensitivity and two isogenic models of acquired resistance to cisplatin and cetuximab, respectively were studied. Cell survival after treatment with CCI-779, cisplatin and cetuximab alone or in combination was determined by MTT assays. Potential predictive biomarkers for tumor cell sensitivity to CCI-779 were evaluated.

**Results:**

We observed considerable heterogeneity in sensitivity of HNSCC cell lines to CCI-779 monotherapy. Sensitivity was observed in *TP53* mutated as well as wild-type cell lines. Total and p-EGFR expression levels but not the basal activity of the mTOR and MAPK signaling pathways were correlated with sensitivity to CCI-779. Resistant cells with increased EGFR activation could be sensitized by the combination of CCI-779 with cetuximab. Interestingly, cell lines with acquired resistance to cisplatin displayed a higher sensitivity to CCI-779 whereas cetuximab-resistant cells were less sensitive to the drug, but could be sensitized to CCI-779 by EGFR blockade.

**Conclusions:**

Activity of CCI-779 in HNSCC cells harboring *TP53* mutations and displaying a phenotype of cisplatin resistance suggests its clinical potential even in patients with dismal outcome after current standard treatment. Cetuximab/mTORi combinations might be useful for treatment of tumors with high expression of EGFR/p-EGFR and/or acquired cetuximab resistance. This combinatorial treatment modality needs further evaluation in future translational and clinical studies.

**Electronic supplementary material:**

The online version of this article (doi:10.1186/s12967-015-0456-6) contains supplementary material, which is available to authorized users.

## Background

Cancer of the head and neck region is the sixth most common cause for cancer-related mortality worldwide with 600,000 new cases and 300,000 deaths per year. Tobacco and heavy alcohol drinking are the most important risk factors [1,2]. Depending on the disease stages, surgery, radiotherapy, and chemotherapy, alone or in combinations, have been used as therapeutic options over the past decades. New treatment strategies such as epidermal growth factor receptor (EGFR) inhibition by cetuximab have been shown to prolong survival, but 5-year survival rates of patients with locally advanced cancers are still at 50% or below [3]. Therefore, new therapeutic approaches like second generation- or multi-target tyrosine kinase inhibitors, proteasome inhibitors, hypoxia-modifying agents, or antiangiogenic agents have been or are being evaluated in clinical trials [4-7]. Among these, one of the most promising approaches is the inhibition of the phosphatidylinositol 3-kinase-related kinase (PI3K) pathway, since it represents the most frequently mutated oncogenic pathway in HNSCC [8].

The mammalian target of rapamycin (mTOR) is a serine/threonine protein kinase and belongs to the PI3K-related kinase protein family. It is involved in many cellular processes like cell growth, proliferation, and survival [9]. Different mTORi including rapamycin, RAD-001 (everolimus) and CCI-779 (cell cycle inhibitor-779, temsirolimus), but also dual mTOR inhibitors that target both of the two mTOR complexes, mTORC1 and mTORC2 have been evaluated [10,11]. In HNSCC, rapamycin [12,13], everolimus [14] and temsirolimus [15], as well as dual inhibitors [16] have already shown promising effects *in vitro* and *in vivo*. Their combination with irradiation, cisplatin or reagents targeting the epidermal growth factor receptor (EGFR) like cetuximab and erlotinib [17,18] has been evaluated in preclinical models [11,19,20] and clinical trials [21]. However, the molecular mechanisms of sensitivity/resistance of HNSCC cells to mTORi remain poorly characterized and no predictive biomarker for patient selection has been established so far.

To evaluate the activity of the mTORi CCI-779 in HNSCC in more detail and to identify predictive biomarkers, the genetic profile, as well as mRNA- and protein expression of genes involved in the mTOR pathway were characterized in 10 HNSCC cell lines and correlated to their sensitivity to CCI-779. Furthermore, the effectiveness of CCI-779 in cells with primary/acquired resistance to cisplatin and cetuximab was evaluated.

## Results and discussion

### Characterization of the growth-inhibitory potential in HNSCC cell lines

**Figure 1:**
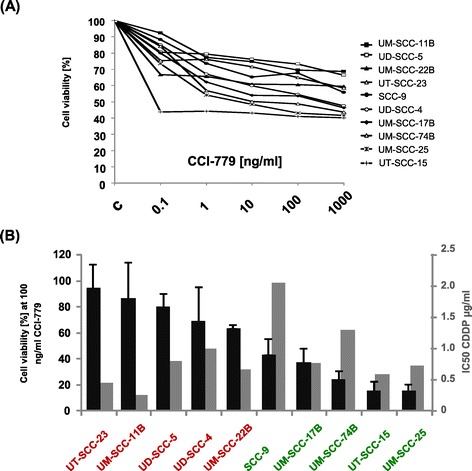
**CCI-779 shows an inhibitory effect on cell survival of cisplatin-sensitive and -resistant HNSCC cell lines. (A)** HNSCC cell lines were treated with increasing doses of CCI-779 (0.1 to 1000 ng/ml). After 72 h cell survival was determined by the MTT assay. **(B)** The same cell lines were treated with CCI-779 at 100 ng/ml for 7 days in long-term MTT assays (black bars, left scale). Their IC50 for cisplatin was as well evaluated in MTT assays (grey bars, right scale). Cells were then grouped in CCI-779-resistant (red) and -sensitive (green) lines using a cutoff of 50% cell survival. (A, B) Survival fractions for given treatments were calculated on the basis of the survival of non-treated cells. Each sample was done in triplicate and experiments were performed thrice.

In all tested HNSCC cell lines treatment with CCI-779 (100 ng/ml) for 72 h reduced cell viability, ranging from a reduction of 69% in UM-SCC-11B cells down to 40% in UT-SCC-15 cells compared to solvent-treated cells (Figure 1 A). In long-term studies (7 days of treatment) we were able to clearly distinguish sensitive from resistant cells, with UT-SCC-23 representing the most resistant (95% viability after treatment with 100 ng/ml) and UM-SCC-25 (16% viability) the most sensitive cell line (Figure 1 B). Of note, the IC50 of cisplatin, a widely used radiosensitizer in HNSCC, only slightly differed between these two cell lines (0.5 and 0.7 μg/ml, respectively), speaking against cross-resistance between the two drugs (Figure 1 B). This finding together with the high efficiency in most of the cell lines tested speaks for CCI-779 as potentially effective therapeutic option in HNSCC cells with primary resistance to cisplatin.

*Neither TP53 status nor mutations in HNSCC-associated oncogenic pathways predict sensitivity to* CCI-779*.*

In search for predictive biomarkers for mTOR inhibition the involvement of *TP53* and other genes from HNSCC-related oncogenic pathways for CCI-779 sensitivity was determined. For this purpose, *TP53* gene and transcript sequences were analyzed by panel next-generation sequencing (NGS) and Sanger sequencing, respectively. In addition, the expression and functional status of the p53 protein was determined. Sequencing revealed distinct mutations of *TP53* in the cell lines tested, with Sanger sequencing and panel NGS giving the same results (Tables 1 and 2). The cyclin-dependent kinase inhibitor 1 (p21) represents one of the p53 targets. Its elevated expression after irradiation served as a readout for functional activity of p53. There was no significant correlation observed between the expression of p53 transcripts (p = .988) or proteins (p = .990) or its transcriptional function (p = .607) and the sensitivity of cells to CCI-779 (Table 1). Previously, reduced sensitivity of HNSCC cell lines carrying a *TP53* mutation to a dual PI3K/mTOR inhibitor was reported [22]. In line with this previous study, wt *TP53* was exclusively detected in the group of sensitive cell lines, displaying decreased viability after treatment with 100 ng/ml of temsirolimus compared to mutated cells (mean viability ± SD: wt *TP53* group [N = 3], 0.36 ± 0.19 *vs* mutated TP53 group [N = 7], 0.65 ± 0.27). However, this difference in viability did not reach significance level (p = .139) which might be due to the limited number of cell lines carrying wt *TP53* in our subset.

**Table 1:** **Characteristics of HNSCC cell lines**

**Cell line**	***TP53*** **genotype**	**p53 transcript**	**p53 protein**	**function**	**CCI-779 100 ng/ml**
**UT-SCC-23**	Loss of transcript	**-**	**-**	**-**	**95**
**UM-SCC-11B**	missense mutation	**+**	**+**	**-**	**87**
**UD-SCC-5**	missense mutation	**+**	**+**	**-**	**81**
**UD-SCC-4**	delete, fs (stop codon)	**+**	**-**	**-**	**70**
**UM-SCC-22B**	missense mutation	**+**	**+**	**-**	**64**
**SCC-9**	delete, fs	**+**	**+** (trunc.)	**-**	**44**
**UM-SCC-17B**	wt	**+**	**+**	**+**	**38**
**UM-SCC-74B**	wt	**+**	**+**	**+**	**25**
**UT-SCC-15**	Incorrectly spliced transcript, fs	**+**	**+**	**-**	**16**
**UM-SCC-25**	wt	**(+)**	**-**	**-**	**16**

Cell lines were ordered by their resistance to CCI-779 at 100 ng/ml. Presented are their *TP53* genotype, transcript- and protein expression. P53 function was assessed by measuring p21 and porphobilinogen deaminase (PBGD) mRNA expression before and after irradiation, using a cut-off of 1.5 fold induction.

**Table 2:** **Mutations identified by panel next-generation sequencing for cell lines (upper panel) and resistance models (lower panel) used in this study**

**Cell line**	**Cell cycle control**	***TP53***	**Cell death regulation**	**RTK signaling**	**PI3K-akt signaling**	**MAPK-signaling**	**Others**
**UT-SCC-23**		**DEL**					***SMAD4,*** *FAT1*
**UM-SCC-11B**		**Cys110Ser**					
**UD-SCC-5**		**His47Tyr**		*ERBB4, PDGFR*			*FAT1, PCDH15*
**UD-SCC-4**		**DEL**		*KDR, MYC*			*FAT1, PCDH15*
**UM-SCC-22B**		**Tyr88Cys**	*CASP8*	*KIT*			***NOTCH1, SMAD4,*** *FAT1, PCDH15,*
**SCC-9**		**Val142fs**		***KDR, RET***			*FAT1, Notch1, PCDH15*
**UM-SCC-17B**				*EGFR, KDR, KIT*	*PIK3CA*	*HRAS*	***SMAD4,*** *FAT1*
**UM-SCC-74B**				*KDR, KRAS*			*CDH1, FAT1, NOTCH1, PCDH15*
**UT-SCC-15**	***CDKN2A***	**Arg282Trp**	*CASP8*				*CDH1, FAT1*
**UM-SCC-25**	***CDKN2A***			*ERBB4*			***SMAD4,*** *FAT1*
**FaDu**_**CDDP-S/R**_	*CDKN2A*	**Arg248Leu 2% ➔ 47%**					***SMAD4***
**UD-SCC-4**_CDDP-S/R_		**DEL**		*MYC,* ***KDR 70% ➔ 100%***			*FAT1, PCDH15, NSD1 0% ➔ 38%*
**UT-SCC-9**_CET-S/R_	***CDKN2A***	**DEL**		*MET 35% ➔ 50%*			*FAT1*
**UM-SCC-22B**_CET-S/R_		**Tyr88Cys**	*CASP8*	*KIT*			***NOTCH1, SMAD4,*** *FAT1, PCDH15*

Mutations which affect protein function, as predicted by the SIFT [41] and PolyPhen [42] program are presented in bold, percentages of allele frequency are given if they differed between parental and resistant cells.

Panel NGS revealed further mutations in key oncogenic pathways including receptor tyrosine kinase, PI3K or MAPK signaling in our cell lines (Table 2). Mutations were also found in genes involved in cell cycle control and cell death regulation, as well as in the tumor suppressor *SMAD4* and the transmembrane receptor gene *NOTCH1*. None of these mutations was associated with sensitivity to CCI-779. Since only one cell line within our panel carried a *PI3KCA* mutation, the involvement of this alteration in sensitivity to mTORi, as discussed in other studies [8,22], could not be addressed.

*CCI-779 sensitivity does not correlate with mTOR/MAPK signaling pathway activity.*

**Figure 2:**
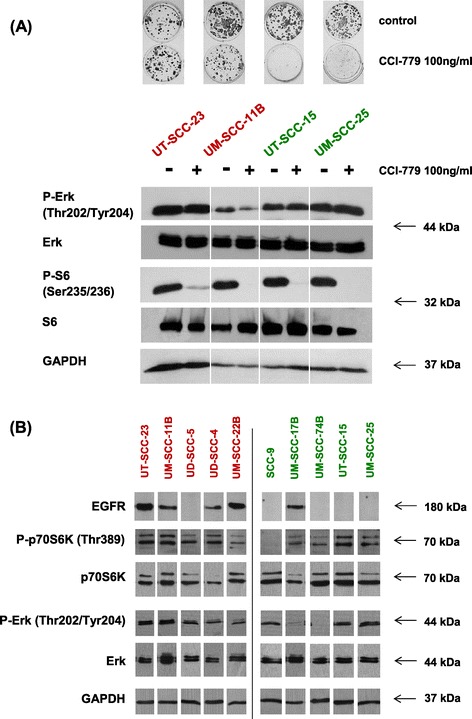
**Resistance to CCI-779 is associated with high EGFR expression but does not correlate with reduced activity of CCI-779 in blocking mTOR-signaling. (A)** Two resistant (red, UT-SCC-23 and UM-SCC-11B) and two sensitive (green, UT-SCC-15 and UM-SCC-25) cell lines were treated with 100 ng/ml CCI-779 for 24 h. The activation of the MAPK- (p-Erk) and mTOR- (p-S6) signaling pathways, as well as the expression of the housekeeper protein GAPDH were evaluated using Western Blot. **(B)** All 10 cell lines were analyzed regarding their basal expression levels of EGFR, p-Erk/Erk (MAPK pathway), and p-S6K/S6K (mTOR pathway) by Western Blot.

As potential biomarker for mTORi activity, the basal activation of the mTOR or MAPK-pathway has been discussed as well as the ability of mTORi to inhibit downstream signaling of mTOR. It is thought that high activity of the mTOR pathway renders tumor cells more dependent on mTOR and results in higher sensitivity to mTORi, whereas high activity of MAPK signaling has been associated with reduced sensitivity to mTOR inhibition [8,23,24]. We therefore evaluated basal protein expression levels of p-S6 (a downstream target of mTOR) and p-Erk (a member of the MAPK pathway) as well as their expression levels after mTOR inhibition in four cell lines with highest and lowest sensitivity to CCI-779 (Figure 2 A). No correlation could be found between sensitivity of cells to CCI-779 and their basal expression levels of p-S6 or p-Erk, with all cell lines expressing phosphorylated, activated forms of these kinases. Furthermore, CCI-779 was able to inhibit phosphorylation of S6, downstream of p70S6K in all tested cell lines regardless of their sensitivity to the drug in the proliferation assays (Figure 2 A). To validate these findings, we included all cell lines used in the proliferation assays. Again, no correlation was found between basal expression of total or activated proteins of Erk/p-Erk or p70S6K/p-p70S6K and sensitivity to CCI-779 (Figure 2 B).

*HNSCC cells with primary resistance to CCI-779 display high EGFR expression and can be sensitized by EGFR blockade.*

**Figure 3:**
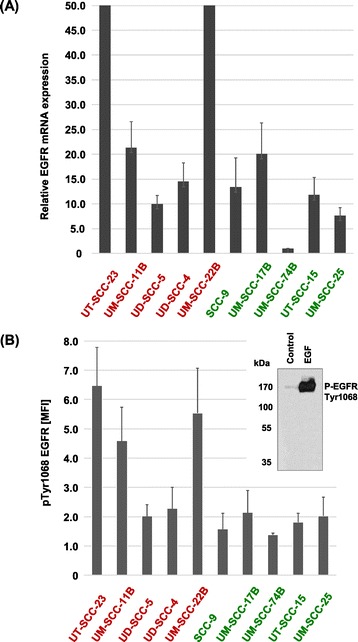
**Cell lines resistant to CCI-779 demonstrate higher expression levels of EGFR mRNA and elevated p-EGFR protein levels. (A)** EGFR mRNA levels were measured by Real Time RT-PCR and correlated to the housekeeper gene Tubulin alpha-6. The relative EGFR mRNA expression was calculated using UM-SCC-74B with the lowest EGFR expression levels as reference and is given as fold levels. **(B)** To quantify the basal expression of phosphorylated, activated EGFR (p-EGFR Tyr1068) flow cytometry was used. Results are given as the median specific fluorescence intensity (MFI) of cells stained with the specific antibody divided by the intensity of cells stained with the isotype control antibody.

**Figure 4:**
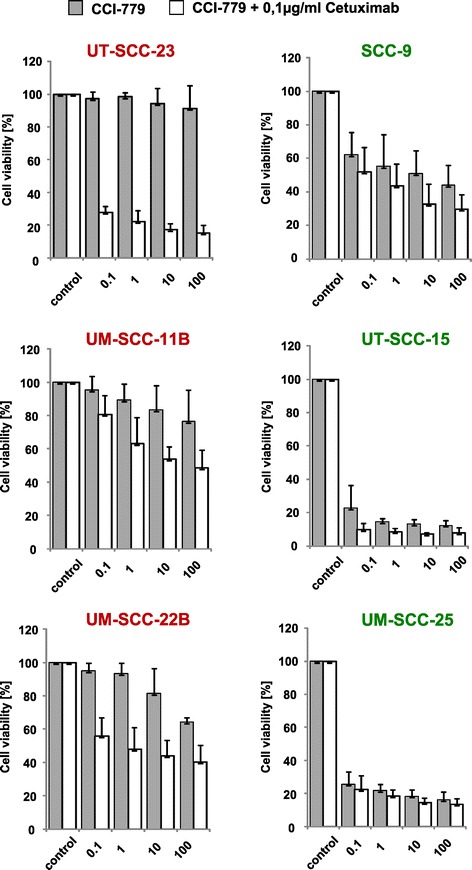
**The combination of CCI-779 and cetuximab augments the sensitivity of cells to treatment with the mTOR inhibitor alone.** All 10 cell lines were treated with increasing doses of CCI-779 (0.1-100 ng/ml) and long-term cell viability (7 days) was measured using the MTT assay. Additionally, CCI-779 treatment was combined with cetuximab at a low dose (0.1 μg/ml), at which the drug itself showed only little effect. In three EGFR-expressing, CCI-779 resistant cell lines the combination led to decreased cell viability (left graphs). Cell viability of sensitive cells could not further be decreased by the combination (three representative cell lines, right graphs). Survival fractions after drug treatment were calculated on the basis of the survival of non-treated cells.

High EGFR protein and mRNA expression were detected almost exclusively in resistant cells (Figures 2 B and 3 A). Interestingly, p-EGFR expression was elevated in the group of cell lines with reduced sensitivity to CCI-779 (Figure 3B; mean p-EGFR expression ± SD: resistant group [N = 5], 4.18 ± 1.97 vs sensitive group [N = 5], 1.78 ± 0.29). Statistical analysis revealed borderline significance (p = 0.052) for this association. Basal MAPK- (p-Erk) and mTOR- (p-p70S6K) pathway activity did not differ between resistant and sensitive cells, therefore other downstream targets of EGFR might be involved in resistance to CCI-779. To test the hypothesis that overexpression of p-EGFR contributes to resistance to CCI-779, three resistant cell lines, expressing high levels of EGFR and p-EGFR, and three sensitive lines, expressing low EGFR/p-EGFR levels, were treated with increasing concentrations of CCI-779 alone or in combination with a fixed low dose of cetuximab. All of the resistant cell lines could be sensitized to CCI-779 by the combination whereas no further sensitization of the sensitive cell lines was observed (Figure 4). The greatest effect was seen in the most resistant cell line UT-SCC-23 with the highest EGFR/p-EGFR expression. Cell viability after CCI-779 treatment was reduced from 91% to 15% after combining CCI-779 with cetuximab. This finding suggests that patients with high EGFR/p-EGFR expressing tumors could benefit from a combined mTORi/anti-EGFR treatment. In support of these results, targeting of mTOR and EGFR in preclinical models of HNSCC has previously been shown to result in a higher growth inhibition than treatment with each agent alone [18,25,26]. Several studies have already been initiated for the identification of biomarkers of mTORi efficacy, with a focus on activation of the mTOR and the MAPK pathway before or after treatment (NCT00195299, CCI-779; NCT01195922, rapamycin; both advanced disease). Moreover, EGFR expression among others is being evaluated as biomarker for RAD001 activity in refractory recurrent locally advanced HNSCC (NCT01051791).

In our model of acquired cetuximab resistance, we showed that cetuximab-resistant cell lines either did not change their sensitivity or became more resistant to CCI-779 (temsirolimus) compared to their sensitive counterparts. This is in line with results from the PII MAESTRO study in which metastatic HNC patients with documented progression on cetuximab were treated with either temsirolimus alone or its combination with cetuximab [27]. This study revealed modest and short activity of temsirolimus as single agent in this scenario. The addition of temsirolimus to cetuximab was able to overcome acquired cetuximab resistance in only a small subgroup of patients (12.5%), however, these responses were more durable than the response to the previous cetuximab-containing regimens [27]. In our model of acquired cetuximab resistance, the combined treatment of cetuximab-resistant cells with cetuximab and CCI-779 was more effective than CCI-779 alone, supporting potential clinical value of this combination. It will be important to evaluate in future studies whether expression levels of EGFR/p-EGFR which we established here as predictive factor for low CCI-779 sensitivity in cetuximab-naïve HNSCC cells might also be useful as predictive biomarker for clinical benefit of cetuximab/temsirolimus combinations in the cetuximab-refractory setting.

*HNSCC cells with acquired resistance to cisplatin or cetuximab show differential sensitivity to* CCI-779 *and can be resensitized by combinatorial treatment.*

**Figure 5:**
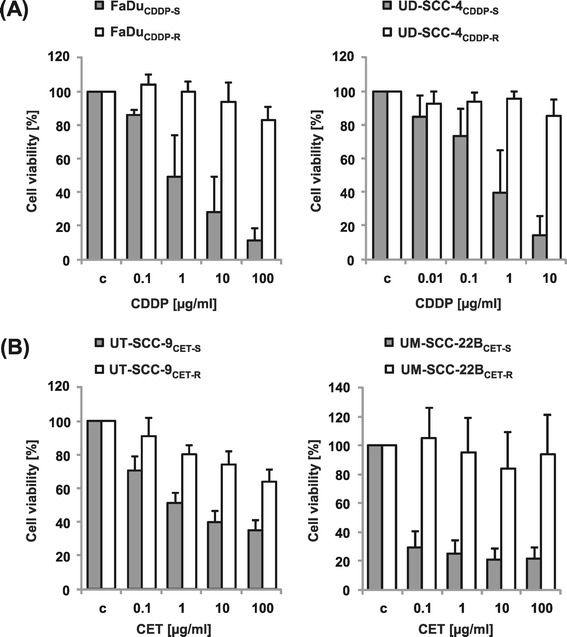
**Long-term treatment of HNSCC cells with cisplatin or cetuximab selects resistant subclones.** Cell lines were treated for 6-9 months with increasing doses of **(A)** cisplatin (FaDuCDDP-S, UD-SCC-4CDDP-S) or **(B)** cetuximab (UT-SCC-9CET-S, UM-SCC-22BCET-S). Doses were elevated if the cells showed a stable growth. The resulting resistant cells (CDDP-/CET-R) as well as their sensitive parental counterparts (CDDP-/CET-S) were used forfurther experiments.

**Figure 6:**
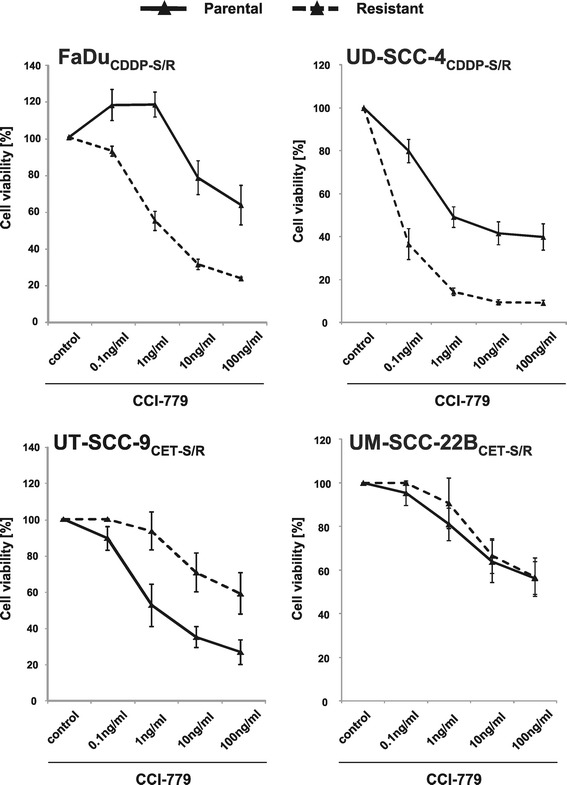
**Cell lines with acquired cisplatin resistance become more sensitive to CCI-779 whereas acquired cetuximab resistance leads to decreased sensitivity to CCI-779.** The sensitivity of cisplatin-resistant cells (upper panels, dashed lines) is increased when compared to their parental counterparts (upper panels, solid lines). In contrast, cetuximab-resistant cells (lower panels, dashed lines) when compared to the parental cells (lower panels, solid lines) remain resistant to CCI-779 (UM-SCC-22B_CET-R_) or become more resistant to the drug (UT-SCC-9_CET-R_). The survival fractions shown were evaluated with long-term MTT assays and calculated on the basis of the survival of non-treated cells.

Cisplatin and cetuximab, each of them in combination with radiotherapy, are effective drugs in the primary treatment of locally advanced HNSCC. In the recurrent disease setting the tumor response to these agents is reduced, most likely due to selection of drug-resistant cells during primary treatment. To evaluate the potential of CCI-779 in the recurrent disease, we established four models of acquired resistance to cisplatin (FaDu and UD-SCC-4) and cetuximab (UT-SCC-9 and UM-SCC-22B) by long-term treatment of HNSCC cell lines with increasing concentrations of the drugs (Figure 5 A and B). Interestingly, we observed that both cell lines with acquired cisplatin resistance (FaDu_CDDP-R_ and UD-SCC-4_CDDP-R_) showed a better response to CCI-779 compared to their parental counterparts (FaDu_CDDP-S_ and UD-SCC-4_CDDP-S_). In contrast, cetuximab-resistant cell lines either did not change their sensitivity (UM-SCC-22B_CET-R_) or became more resistant to CCI-779 (UT-SCC-9_CET-R_) compared to the parental cetuximab-sensitive cells (Figure 6).

By panel NGS analysis we detected an increased allele frequency of a specific *TP53* exon mutation (Arg248Leu) in FaDu_CDDP-R_ that was already present in the parental cell line FaDu_CDDP-S_, indicating the selection of a pre-existing subclone (Table 2). In the UD-SCC-4_CDDP-R_ cell line, the selection of subclones harboring *KDR* (*VEGFR2*) and *NSD1* mutations was observed. *TP53* and *KDR* mutations have been associated with cisplatin resistance [28,29] and NSD1 is known to regulate NF-κB [30] which has also been involved in resistance to cisplatin [31]. In one of the two cetuximab-resistant cell lines (UT-SCC-9_CET-R_), we observed the accumulation of a subclone carrying a *MET* mutation which has been shown to be involved in cetuximab resistance [32]. The exact mechanisms of how these genetic alterations are involved in CCI-779 sensitivity have to be elucidated in future studies.

**Figure 7:**
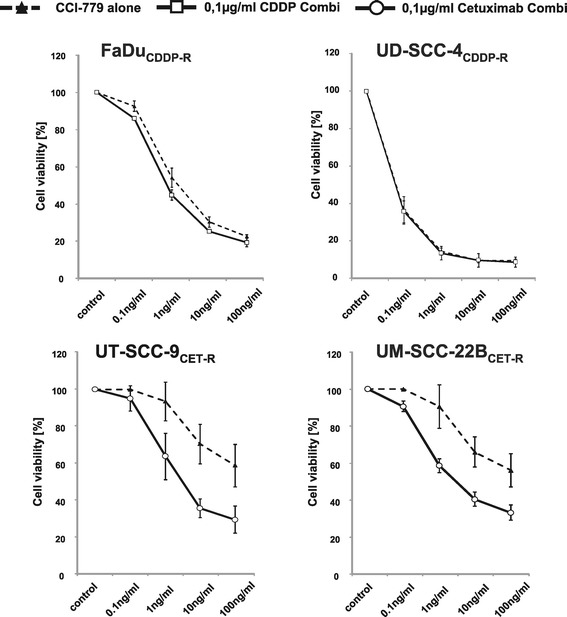
**Addition of CCI-779 to cetuximab potentiates the inhibitory effect on cell survival in HNSCC cells with acquired resistance to cetuximab.** Cell viability of cisplatin-resistant cells (upper panels, dashed lines) can only be slightly increased in the FaDu_CDDP-R_ model when combining CCI-779 with cisplatin (solid lines) compared to CCI-779 single treatment. In cetuximab-resistant cells (lower panels, dashed lines), the addition of cetuximab to CCI-779 further decreases cell viability in both models (solid lines).

We next assessed if combinatorial treatment with CCI-779 and cisplatin or cetuximab increased growth inhibition in these cell line models of acquired drug resistance. Cisplatin-resistant cells with increased sensitivity to CCI-779 could be only slightly sensitized further by addition of cisplatin to CCI-779 only in the FaDu but not in the UD-SCC-4 model (Figure 7). Cetuximab-resistant cells with reduced sensitivity to CCI-779 could be sensitized to CCI-779 by its combination with cetuximab in both cell line models of acquired cetuximab resistance (Figure 7).

These data suggest that patients with recurrent cisplatin-resistant tumors might benefit from mTORi treatment. In line with our results, a clinical trial in recurrent/metastatic HNSCC addressing the efficacy of temsirolimus after cisplatin treatment failure has shown promising results [33]. Further drug partners in this setting will have to be identified given the absence of any potentiating effects of the CCI-779/cisplatin combination. Treatment with the combination of cetuximab and mTORi, but not mTORi alone could be beneficial for patients with tumors with acquired cetuximab-resistance. Such dual inhibition of EGFR and the mTOR pathway is currently being tested and our study supports the need for further prospective trials in HNSCC.

## Conclusions

The results from our study demonstrating no cross-resistance of HNSCC cells to CCI-779 and cisplatin suggests that mTORi could serve as a potential treatment option in tumors with primary resistance to cisplatin. We identified EGFR/p-EGFR expression as potential negative predictive biomarker for mTORi efficacy and the combination of CCI-779 and cetuximab as a therapeutic regimen with increased growth-inhibitory potential. Our results from the preclinical models of acquired resistance also suggest clinical potential of mTORi in the recurrent, cisplatin-refractory setting and its combination with cetuximab in tumors with acquired resistance to EGFR blockade.

## Methods

### Cell lines and reagents

The HNSCC cell lines UD (University of Düsseldorf)-SCC-4, -5, UT (University of Turku)-9, -15, -23, UM (University of Michigan)-SCC-11B, -17B, -22B, -25, -74B, and SCC-9 were a gift from T.K. Hoffmann (University of Essen) and T.E. Carey (University of Michigan) [34]. FaDu was purchased from ATCC. The identity of the cell lines was confirmed by high-throughput SNP-based authentication (Multiplexion, Heidelberg, Germany). All cell lines were tested for mycoplasma at monthly intervals by RT-PCR [35]. Contaminated cultures were treated with Mycoplasma Removal Agent (MP Biomedicals, Santa Ana, USA) according to the manufacturer’s protocol. Cells were cultured in Minimal Essential Medium (MEM) supplemented with 10% heat-inactivated fetal bovine serum and 1× non-essential amino acids. Acquired resistance to cisplatin or cetuximab was attained by culturing the cells for a period of 4-9 months in increasing concentrations of the respective drug. All cell culture reagents were from GIBCO (life technologies, Carlsbad, CA, US). Cell cultures were incubated at 37°C and 5% CO2 in a humidified atmosphere. CCI-779 was provided by Pfizer (Berlin, Germany) and diluted in dimethylsulfoxide (DMSO) to a stock of 1 mg/ml. Cisplatin was purchased from Sigma-Aldrich (Munich, Germany) and cetuximab was provided by Merck (Darmstadt, Germany). Working solutions were freshly prepared from the stock solution by dilution in cell culture medium on the day of the experiment.

### MTT viability assays

Cells were seeded into 96-well plates at a density of 250-300 cells/well. Twenty-four hours after seeding, cells were treated with CCI-779, cisplatin, cetuximab, or the combination. Cells were then incubated for 72 h (short-term) or 7 days (long-term). At the end of the experiments 3-(4,5-dimethylthiazol-2-yl)-2,5-diphenyltetrazolium bromide reagent (MTT) was added to the cells and after one hour incubation formazan complexes were dissolved in DMSO and absorbance measured with a spectrophotometer. Survival fractions for given treatments were calculated on the basis of survival of untreated cells. Each sample was done in sextuplets and at least three independent experiments were carried out. From the dose-effect curves the IC50 values for cisplatin were calculated.

### Immunoblotting analysis

Expression levels of phosphorylated and total proteins in cell lysates of HNSCC cell lines, either untreated or treated with 100 ng/ml CCI-779 were assessed by standard Western blot analysis. Briefly, cells were harvested by scraping in RIPA buffer. Standard SDS–polyacrylamide gel electrophoresis was performed using 40 μg of total protein per sample, followed by transfer to PVDF membranes (Millipore, Billerica, MA, US). For detection, the following antibodies were used: p-EGFR Tyr1068 (#2234), p-Erk Thr202/Tyr204 (clone D13.14.4E), Erk (clone 137 F5), p-S6 Ser235/236 (clone D57.2.2E), S6 (clone 5G10), p-S6K Thr389 (clone 108D2), S6K (clone 49D7) and GAPDH (clone 14C10), all from Cell Signaling Technology (Danvers, MA, US). The antibody against p53 (clone DO-1) was purchased from Santa Cruz (Santa Cruz, CA, US) and anti-EGFR (clone 13) from BD Biosciences (Franklin Lakes, NJ, US). Secondary antibodies included peroxidase-conjugated goat anti-mouse and goat anti-rabbit both from Jackson ImmunoResearch Laboratories (West Grove, PA, US). The immunoreactivity was detected using the Pierce ECL Plus Western Blotting Substrate (Thermo Scientific, Waltham, MA, US).

### Flow cytometry

Cells were harvested by trypsinization and fixed in fixation buffer (Nordic MUbio, Susteren, Netherlands). Cells were incubated with primary and secondary antibodies in permeabilization buffer (Nordic MUbio) for 20 min each and were subsequently analyzed using the FACSCanto II cytometer and the FACSDiva Software v6 (BD Biosciences). The following antibodies were used for staining: rabbit anti-p-EGFR Tyr1068 (cell signaling), rabbit IgG isotype control and goat anti-rabbit FITC (Life Technologies, Darmstadt, Germany). Median specific staining intensity (MFI) was calculated as ratio of the fluorescence intensity of cells stained with the specific antibody and the isotype control.

### Sequencing analysis

RNA was isolated and TP53 transcript expression analysis as well as Sanger sequencing was performed (Source BioScience, Berlin, Germany). For the panel-based next generation sequencing experiments, library preparation and semiconductor sequencing was performed as follows. Using the multiplex PCR based Ion Torrent approach as described previously [36,37], for this study an in-house gene panel for 45 HNSCC-related cancer genes was designed (see Additional file 1) using the COSMIC database [38] as well as two publications on exome sequencing data of HNSCC tumor material [39,40]. DNA was extracted from all of the cell lines with the Invisorb Spin Tissue Mini Kit (Stratec, Birkenfeld, Germany). Amplicon library preparation was performed using approximately 10 ng of DNA as advised by the manufacturer. Briefly, the DNA was mixed with the primer pool, containing all primers for generating the 224 amplicons and the AmpliSeq HiFi Master Mix, and transferred to a PCR cycler (BioRad, Munich, Germany). PCR cycling conditions were as follows: Initial denaturation: 99°C for 2 min, cycling: 21 cycles at 99°C for 15 sec and 60°C for 4 min. Subsequent to the PCR reaction, primer end sequences were partially digested using the FuPa reagent as instructed, followed by the ligation of barcoded sequencing adapters (Ion Xpress Barcode Adapters, Life Technologies). The final library was purified using AMPure XP magnetic beads (Beckman Coulter, Krefeld, Germany) and quantified using qPCR (Ion Library Quantitation Kit, Life Technologies) on a StepOnePlus Instrument (Life Technologies). The individual libraries were diluted to a final concentration of 100pM and eight to ten libraries were pooled and processed to library amplification on Ion Spheres using the Ion OneTouch 2 instrumentation with the 200 bp chemistry. Unenriched libraries were quality-controlled using Ion Sphere quality control measurement on a QuBit instrument. After library enrichment (Ion OneTouch ES), the library was processed for sequencing using the Ion Torrent 200 bp sequencing chemistry and the barcoded eight to ten libraries were loaded onto a single 318 chip.

Raw data analysis was performed using Ion Torrent Software Suite (Version 3.6 and 4.0). The reads were aligned to the human reference sequence build 38 (hg19) using the TMAP aligner implemented in the Torrent Suite software. Detection of single base pair variants and insertion-deletion polymorphisms (InDels) compared to the human reference sequence was performed using either Ion Torrent Variant Caller (3.6 and 4.0). Detection thresholds for SNPs and InDels were set at an allele frequency of 5%. Variants were annotated and filtered against the dbSNP and COSMIC databases and screened for possible splice site effects using the CLC genomics Suite 6 (CLCbio, Aarhus, Denmark). Copy number variations were determined using the coverage analysis plug-in of the Torrent Suite software.

### Transcript expression analysis

Basal and irradiation-induced p21 expression levels, as read-out for p53 transcriptional activity in HNSCC cell lines, as well as basal EGFR levels were determined by quantitative reverse-transcriptase polymerase chain reaction (qRT-PCR). Total cellular RNA extraction was performed using the High Pure RNA Isolation Kit (Roche, Basel, Switzerland). Synthesis of cDNA was done with the Omniscript Reverse Transcription kit (QIAGEN, Hamburg, Germany), according to the supplied protocol, using random hexamers and oligo dT15 primers (Roche) and 2 μg of total RNA. The quality of RNA was checked by GAPDH PCR and only samples positive for GAPDH transcripts were used for analysis. Real-time-PCR was performed in a reaction volume of 20 μl containing 2 μl cDNA, Light Cycler TaqMan Master (Roche), primers and probes for p21, EGFR and the housekeeping gene porphobilinogen deaminase (*PBGD*) in concentrations recommended by the manufacturer (Real Time Ready Assays, Roche). PCR cycling was performed using a Light Cycler (Roche). Relative quantification of p21 and EGFR expression was done by normalization to the expression levels of PBGD.

### Statistical analysis

All statistical analyses were performed using SPSS v.20.0 (IBM Corp., Armonk, NY, USA) software. The significance of differences in the cell survival fraction after treatment with 100 ng/ml temsirolimus of cell lines exhibiting diverse characteristics regarding *TP53* was determined using the independent-samples *t*-test. The level of significance was set at p < 0.05.

## Additional file

Additional file 1:
**HNSCC-Panel.**
