# Supplementary material for: CCI-779 (Temsirolimus) exhibits increased anti-tumor activity in low EGFR expressing HNSCC cell lines and is effective in cells with acquired resistance to cisplatin or cetuximab
Source: J Transl Med. 2015 Apr 1;13:106. doi: 10.1186/s12967-015-0456-6 (PMC4389307; doi:10.1186/s12967-015-0456-6)
Supplement: Additional file 1: — HNSCC-Panel. [file 12967_2015_456_MOESM1_ESM.pdf]

**AKT1**<br>(3, 6)	**ERBB4**<br>(3,4,6,7,8,9,15,23)	**KRAS**<br>(2,3)	**PTEN**<br>(1,3,5,6,7,8)
**ALK**<br>(23, 25)	**FAT1**<br>(2,6,10,19,25)	**LRP1B**<br>(11,42,81)	**PTPN11**<br>(3,13)
**BRAF**<br>(11,15)	**FBXW7**<br>(5,8,9,10,11)	**MET**<br>(2,11,14,16,19)	**RB1**<br>(2,3,6,11,13,16,17,18,20,21,22,23)
**CASP8**<br>(3,9,10)	**FGFR1**<br>(5,7,10,14)	**MLL2**<br>(11,48)	**RET**<br>(10,11,13,15,16)
**CCND1**<br>(1,3)	**FGFR2**<br>(7,9,12)	**MYC**<br>(2,3)	**SMAD4**<br>(3,4,5,6,8,9,10,11,12)
**CCNE1**<br>(5,10)	**FGFR3**<br>(7,9,14,16,18)	**NFE2L2**<br>(2)	**SMO**<br>(3,5,6,9,11)
**CDH1**<br>(2,3,4,5,6,7,9,10,11,12,13,14,16)	**FLT3**<br>(11,14,16,20)	**NOTCH1**<br>(3,6,7,8,12,15,18,20,22,23,25,26,27,32,34)	**SRC**<br>(14)
**CDKNA2**<br>(1,2)	**HRAS**<br>(2,3)	**NRAS**<br>(2,3,4)	**STK11**<br>(1,2,4,5,6,8)
**CSMD3**<br>(5,14,63,65)	**JAK2**<br>(12,14,25)	**NSD1**<br>(5,14,19)	**TP53**<br>(4,5,6,7,8,9,10)
**CTNNB1**<br>(3,5,9)	**JAK3**<br>(4,13,16)	**PCDH15**<br>(11,14,19,27)	
**EGFR**<br>(3,7,15,18,19,20,21)	**KDR**<br>(6,7,11,19,21,26,27,30)	**PDGFRA**<br>(5,10,11,12,14,15,18)	
**ERBB2**<br>(19,20,21)	**KIT**<br>(2,9,10,11,13,14,15,17,18)	**PIK3CA**<br>(2,5,8,10,14,21)	

HNSCC-Panel

- 45 Gene (exons covered), 224 Amplicons
